# Stereotactic Body Radiotherapy as Primary Treatment for Elderly Patients with Medically Inoperable Head and Neck Cancer

**DOI:** 10.3389/fonc.2014.00214

**Published:** 2014-08-11

**Authors:** John A. Vargo, Robert L. Ferris, David A. Clump, Dwight E. Heron

**Affiliations:** ^1^Department of Radiation Oncology, University of Pittsburgh Cancer Institute, Pittsburgh, PA, USA; ^2^Department of Otolaryngology, Division of Head and Neck Surgery, University of Pittsburgh Cancer Institute, Pittsburgh, PA, USA

**Keywords:** SBRT, cetuximab, elderly, head and neck cancer, radiosurgery

## Abstract

**Purpose**: With a growing elderly population, elderly patients with head and neck cancers represent an increasing challenge with limited prospective data to guide management. The complex interplay between advanced age, associated co-morbidities, and conventional local therapies, such as surgery and external beam radiotherapy ± chemotherapy, can significantly impact elderly patients’ quality of life (QoL). Stereotactic body radiotherapy (SBRT) is a well-established curative strategy for medical-inoperable early-stage lung cancers even in elderly populations; however, there is limited data examining SBRT as primary therapy in head and neck cancer.

**Material/methods**: Twelve patients with medically inoperable head and neck cancer treated with SBRT ± cetuximab from 2002 to 2013 were retrospectively reviewed. SBRT consisted of primarily 44 Gy in five fractions delivered on alternating days over 1–2 weeks. Concurrent cetuximab was administered at a dose of 400 mg/m^2^ on day −7 followed by 250 mg/m^2^ on day 0 and +7 in *n* = 3 (25%). Patient-reported quality of life (PRQoL) was prospectively recorded using the previously validated University of Washington quality of life revised (UW-QoL-R).

**Results:** Median clinical follow-up was 6 months (range: 0.5–29 months). The 1-year actuarial local progression-free survival, distant progression-free survival, progression-free survival, and overall survival for definitively treated patients were 69, 100, 69, and 64%, respectively. One patient (8%) experienced acute grade 3 dysphagia and one patient (8%) experienced late grade 3 mucositis; there were no grade 4–5 toxicities. Prospective collection of PRQoL as assessed by UW-QoL-R was preserved across domains.

**Conclusion**: Stereotactic body radiotherapy shows encouraging survival and relatively low toxicity in elderly patients with unresectable head and neck cancer, which may provide an aggressive potentially curative local therapy while maintaining QoL.

## Introduction

With a growing elderly population expected to exceed 80,000,000 in the United States by the year 2050, the incidence of elderly patients with head and neck cancers is similarly expected to drastically increase with a projected incidence over 31,000 by the year 2030 (1, 2). Elderly patients with head and neck cancers represent a clinical challenge with limited prospective data to guide management, as patients over 65–70 are often excluded from the randomized trials that shape management (3). Elderly patients more commonly present with locally advanced disease with less neck involvement, highlighting the potential opportunity of aggressive local therapy (4, 5). However, the complex interplay between advanced age, associated co-morbidities, and conventional local therapies such as surgery and external beam radiotherapy ± chemotherapy, can carry significant impact on elderly patients’ quality of life (QoL) (6). Increasing age and co-morbidity can increase risks of treatment-related complications and compromise outcomes. The potential negative impact of increasing age on treatment outcomes was well delineated in the MACH meta-analysis, where chemotherapy resulted in an absolute improvement of 6.5% in 5-year overall survival for all patients but there was no overall survival benefit for the addition of chemotherapy to definitive radiotherapy in patients > 70 years of age (7).

Cetuximab, a humanized murine monoclonal antibody against the epidermal growth factor receptor, has been shown to improve overall survival over radiotherapy alone and is an attractive systemic therapy in elderly patients that potentially avoids the oto- and nephrotixicty as well as mucositis common to platinum-based regiments used in head and neck cancer (8, 9). Similarly, stereotactic body radiotherapy (SBRT) is an advanced radiation planning and delivery technique that delivers a highly focused radiation dose per fraction (≥6 Gy) in 1–5 fractions and is a well-established curative strategy for medical-inoperable early-stage lung cancers especially in elderly populations (10). SBRT ± cetuximab has emerged as a promising salvage strategy for unresectable locally recurrent previously irradiated squamous-cell carcinomas of the head and neck (11–14). When compared to conventionally fractionated external beam radiotherapy, primary benefits of short overall treatment time (five fractions over 1–2 weeks) and minimal acute toxicity makes SBRT ± cetuximab a potentially attractive treatment strategy in elderly patients. We hypothesize that primary SBRT ± cetuximab may provide a similarly effective local therapy that minimizes acute toxicity and overall treatment time for elderly patients with medically inoperable well-lateralized head and neck cancers.

## Materials and Methods

**Figure 1:**
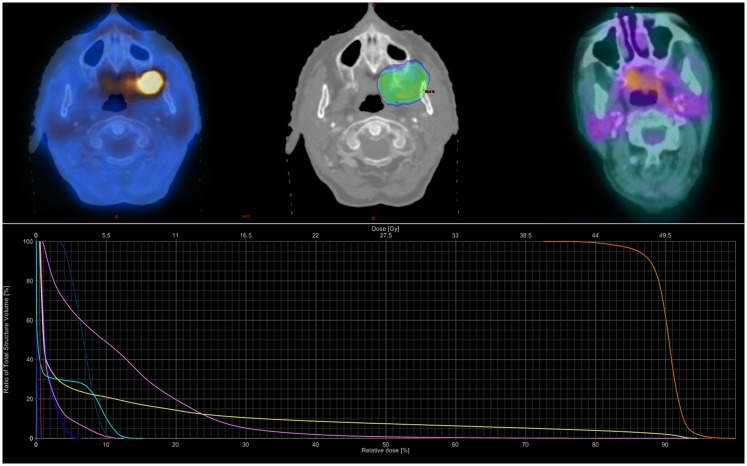
**Sample SBRT treatment plan**. Case example for an 88-year-old female with a T4aN0M0 squamous-cell carcinoma of the left buccal mucosa. She received 44 Gy in five fractions prescribed to the 80% isodose line over 10 elapsed days using the TrueBeam^®^ RapidArc™ platform and three non-coplanar arcs. Dose–volume histogram shows the PTV (orange), oral cavity (pink), mandible (yellow), spinal cord (chartreuse), and parotids (right dark blue, left blue-green). She completed therapy with but grade 1 mucositis; she later developed grade II oral ulceration and trismus. She remained NED with complete metabolic response until dying from co-morbidities 20 months following SBRT.

Following Institutional Review Board approval, a retrospective review (2002–2013) identified 12 patients of advanced age (median age 88 years) with medically inoperable head and neck cancer treated with SBRT ± cetuximab. Patients were selected for a primary radiosurgical approach on a case-by-case basis at the discretion of a multidisciplinary head and neck tumor board; generally patients were selected based on a well-lateralized lesion and concern for an inability to tolerate or patient refusal of conventional treatment regimes. Following prior phase I dose-escalation study in the re-irradiation setting, SBRT consisted primarily of 44 Gy in five fractions delivered on alternating days over 1–2 weeks (see Figure 1) (13). Spinal cord doses was constrained to not exceed 8–10 Gy (with cumulative maximum of 50 Gy for those receiving prior radiotherapy), while the remaining normal tissues be constrained as much as possible without compromising the target volume on a case-by-case determination. SBRT was delivered via the CyberKnife^®^, Trilogy^®^, or TrueBeam^®^ platforms with custom thermoplastic mask immobilization and daily image guidance either via X-Sight^®^ skull tracking, daily cone beam CT, or BrainLab ExacTrac^®^. Early in our radiosurgery program, the gross tumor volume (GTV) was equal to the planning target volume (PTV), following a deformable registration analysis of the patterns of failure following SBRT; since 2012, we have employed a 2–5 mm GTV to PTV expansion (15). Concurrent cetuximab was administered at a dose of 400 mg/m^2^ on day -7 followed by 250 mg/m^2^ on day 0 and +7 in *n* = 3 (25%).

Patient-reported quality of life (PRQoL) was prospectively recorded using the previously validated University of Washington quality of life revised (UW-QoL-R) as part of an institutionally maintained database (16). UW-QoL-R measures QoL in 12 domains specific to head and neck cancer and three domains of global health status using a single Likert-type question with an assigned score of 0–100 (100 representing normal function). UW-QoL-R surveys were administered at initial consultation and each subsequent follow-up appointment, usually 1-month post-irradiation then every 3 months. Mean scores and standard deviations (SD) were calculated from UW-QoL-R and compared to baseline values using the Wilcoxon signed-rank test. Toxicity was physician record as per National Cancer Institute Common Terminology Criteria for Adverse Events version 4.0 (CTCAE v4). Survival and tumor control were estimated using the Kaplan Meier method using SPSS Version 21 (SPSS, Chicago, IL, USA) calculated from the time of SBRT to the date of failure or last follow-up/death. Patients treated for palliative intent with metastatic disease prior to radiosurgery were excluded for survival and tumor control analysis, but were included for toxicity and QoL assessments.

## Results

Baseline patient characteristics are outlined in Table 1. Briefly, the median age at the time of radiosurgery was 88 years, with 57% female. The most common primary sites were oral cavity (25%) and salivary gland/paranasal sinus (25%). Sixty-seven percent were AJCC stage IVA, with a median treatment volume of 42.1 cc. Three patients (25%) were treated for local recurrence following initial surgery with no prior radiation therapy. No patients completed prior (full dose) definitive chemoradiation; however, two patients (17%) terminated conventional external beam radiotherapy + cetuximab after 12 and 30 Gy. The interval between conventional external beam radiotherapy and SBRT for these patients was 1 month and 2 years. Ninety-two percent completed the prescribed course without major treatment interruption, with one patient (8%) terminating treatment after four of a planned five fractions due to declining performance status.

**Table 1:** **Baseline patient characteristics**.

Baseline characteristics	All patients (*n* = 12)
Concurrent cetuximab	
SBRT + cetuximab	3 (25%)
SBRT alone	9 (75%)
Age (years), median (range)	88 (79–98)
Gender	
Male	5 (42%)
Female	7 (58%)
Primary site
Larynx	1 (8%)
Nasopharynx	1 (8%)
Oropharynx	2 (17%)
Oral cavity	3 (25%)
Salivary gland/sinuses	3 (25%)
Other	2 (17%)
AJCC stage	
III	2 (17%)
IVA	8 (67%)
IVC	2 (17%)
Tumor volume (cm^3^), median (range)	42.1 (15.1–247.9)
Treatment duration (days), median (range)	10 (1–15)
Palliative intent (M1 disease prior to SBRT)	2 (17%)

Median clinical follow-up was 6 months (range: 0.5–29 months). The median time to death or last follow-up was 16 months (range: 1–33 months). The 1-year actuarial local progression-free survival, distant progression-free survival, progression-free survival, and overall survival for definitively treated patients were 69, 100, 69, and 64%, respectively. Specifics for follow-up and treatment outcomes of the definitively treated cohort are outlined in Table 2. Briefly, of the two patients who experienced a local failure: one was infield and one was an overlap failure. No patients experience isolated neck failure. Of patients who received definitive SBRT, at time of last follow-up, three (30%) were alive without disease, two died with disease (20%), four died without disease recurrence (40%), one (10%) underwent salvage laryngectomy for local recurrence but died of a second primary mucosal melanoma. One patient (8%) experienced acute grade 3 dysphagia and one patient (8%) experienced late grade 3 mucositis; there were no grade 4–5 toxicities. The most common recorded grade 2 toxicities (experienced by > 1 patient) were acute grade 2 mucositis (*n* = 3, 25%), late grade 2 mucosal ulceration (*n* = 3, 25%), and late grade 2 dysphagia (*n* = 2, 17%).

**Table 2:** **Description of definitive treatments and outcomes**.

Age	Primary location	Histology	AJCC stage	PTV (cc)	SBRT total dose (Gy)	Fractions (*n*)	Cetuximab	Local progression (type)	Overall disease status	Time to death or last follow-up (months)
81	Base of tongue	SCC	T4N0M0	26	44	5	Y	N	NED	27
91	Alveolar ridge	SCC	T4N1M0	104	35.2^a^	4^a^	Y	–	DOD	1
86	Parapharnygeal space	NR	T3N0M0	40	25	5	N	Y (overlap)	DOD	22
97	Maxillary sinus	Spindle cell	T3N0M0	53	20	1	N	N	DWOD	33
98	Larynx	SCC	T4N0M0	74	44	5	N	Y (infield, salvaged with laryngectomy)	DWOD	29
88	Buccal mucosa	SCC	T4N0M0	26	44	5	N	N	DWOD	20
87	Parotid	Acinic cell	rT0N2aM0	15	36	6	N	N	DWOD	11
82	Base of tongue	SCC	rT2N0M0 (initial T1N2aM0)	44	44	5	Y	N	DWOD	6
93	Maxillary sinus	SCC	T4N0M0	41	44	5	N	N	NED	5
79	Parotid	Epithelial neoplasm	T4N0M0	248	44	5	N	N	NED	3

*^a^Patient terminated treatment after four fractions of a planned dose of 44 Gy in five fractions due to declining performance status*.

*NED: no evidence of disease; DOD: dead of disease; DWOD: died without evidence of disease progression; NR: not recorded; SCC: squamous-cell carcinoma*.

**Figure 2:**
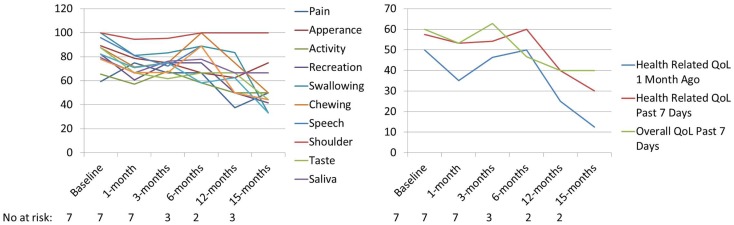
**Mean PRQoL values from baseline to 15 months as assessed by UW-QoL-R**. QoL: quality of life; No. at risk: number of patients at risk.

UW-QoL-R was administered in 92%; with 58% (*n* = 7) completing both pre- and post-SBRT UW-QoL-R surveys. Of patients completing both pre- and post-SBRT UW-QoL-R, the median number of surveys was 3 (range: 2–7 surveys) with a median follow-up survey time of 3 months (range: 0–15 months). At time of last survey, 71% denoted improved or stable overall QoL for the last 7 days as compared to baseline. Over the period of follow-up, there were no significant differences in any of the 12-assessed head and neck specific domains or three domains of global health comparing UW-QoL-R means for patients surviving to 15 months to baseline (see Figure 2).

## Discussion

The results presented here-in for a primary approach of SBRT ± cetuximab show the feasibility in an elderly population with a median age of 88 years. The 1-year local progression-free survival of 69% and overall survival of 64% are comparable to (see Table 3) prior results published for hypofractionated external beam radiotherapy (17–21). Severe toxicity rates were low at 16% overall (8% acute and 8% late toxicity), and 92% of patients were able to complete the prescribed treatment course without interruption or major complication. This overall tolerability of SBRT was perhaps anecdotally best highlighted by the two patients who terminated conventional external beam radiotherapy plus cetuximab but were able to complete SBRT plus cetuximab without interruption. Additionally, prospective collection of patient-report QoL as assessed by UW-QoL-R was preserved. While there were generally negative trends across domains (see Figure 2), comparing baseline to 15-month values these trends did not reach statistical significance. Moreover, at time of last UW-QoL-R survey, the majority of patients (71%) reported improved for stable overall QoL over the last 1 week; consistent with prior reports for QoL outcomes following SBRT for recurrent previously irradiated head and neck cancers (22).

**Table 3:** **Summary of results for hypofractionated conventional external beam radiotherapy in locally advanced head and neck cancer**.

	*n*	Dose	PFS (months)	OS (months)
Porceddu et al. (17)	35	30 Gy in 5 fx	3.9	6.1
Das et al. (18)	33	40 Gy in 10 fx	–	7
Corry et al. (19)	38	14 Gy in 4 fx	3.1	5.7
Al-mamgani et al. (20)	158	50 Gy in 15 fx	14	17
Agarwal et al. (21)	110	40 Gy in 16 fx	1-yr 55%	–
Present study	10^a^	SBRT 20–44 in 1–5 fx	6	15.5

*^a^Progression-free and overall survival rates are only for the 10 definitely treated patients*.

*Fx: fractions; *n*: number of patients; PFS: progression-free survival; OS: overall survival; Gy: Gray; yr: year*.

These results add to a growing yet limited body of prior published data for primary SBRT for patient with medically inoperable head and neck cancer. These series highlight the potential benefits of a primary radiosurgical approach (see Table 4) *vis-a-vis* short treatment time, minimal acute toxicity, and promising local control plus overall survival (23, 24). The integration of cetuximab with primary SBRT is unique to this series. Concurrent cetuximab has been shown to improve progression-free and overall survival when added to conventional fractionated external beam radiation alone and improve outcomes in the recurrent setting when combined with SBRT (11–14). Concurrent cetuximab was well tolerated in conjunction with SBRT for the three patients in our series. However, additional follow-up and data are necessary to better define the potential efficacy when combined with SBRT in the primary setting.

**Table 4:** **Summary of data for primary SBRT in elderly patients**.

	*n*	Dose	LC	OS	Toxicity Grade 3 +
Siddiqui et al. (23)	10	18–48 Gy in 1–8fx	1 yr 83%	1 yr 70%	1 G3 cataract, 1 G3 pain
Kawaguchi et al. (24)	14	35–42 Gy in 3–5fx	71.4% crude	78.6% crude	1 G3 osteonecrosis
Present study	10^a^	20–44 Gy in 1–5fx	1 yr 69%	1 yr 64%	1 G3 dysphagia, 1 G3 mucositis

*^a^Local control and survival rates are only for the 10 definitely treated patients*.

*Fx: fractions; *n*: number of patients; LC: local control; OS: overall survival; G3: grade 3; Gy: Gray; yr: year*.

This series is limited by retrospective design subject to inherent biases, most notably patient selection, and small sample size. While short overall follow-up limits assessment of late complications, this series is strengthened by the addition of prospective collection of PRQoL outcomes. Further prospective studies should evaluate the role of SBRT ± cetuximab as a primary treatment for patient with well-lateralized head and neck cancers that are poor candidates for standard of care combined modality therapy.

## Conclusion

Stereotactic body radiotherapy shows encouraging survival rate and relatively low toxicity in a medically inoperable elderly patients population with head and neck cancer. Treatment was well tolerated in the majority of elderly patients, including those receiving a combination of SBRT plus concurrent cetuximab. SBRT ± cetuximab may provide an aggressive potentially curative local therapy while preserving QoL worthy of further investigation.

## Conflict of Interest Statement

The authors declare that the research was conducted in the absence of any commercial or financial relationships that could be construed as a potential conflict of interest.
